# Effects of Nucleotide-Rich *Kluyveromyces fragilis* and *Saccharomyces cerevisiae* Yeast Extracts on Cognitive Function in Older Adults with Mild Cognitive Impairment: A Randomized Placebo-Controlled Trial

**DOI:** 10.3390/nu18121869

**Published:** 2026-06-10

**Authors:** Hammad Ullah, Marcello Cordara, Maria Vittoria Morone, Roberto Piccinocchi, Lorenza Francesca De Lellis, Angela Cerqua, Alessandra Baldi, Roberto Sacchi, Gaetano Piccinocchi, Alessandro Di Minno, Gaia Spadarella, Maria Daglia

**Affiliations:** 1 Department of Pharmacy, COMSATS University Islamabad, Abbottabad Campus, Abbottabad 22060, Pakistan; hammadrph@gmail.com; 2 School of Medicine, University of Milano-Bicocca, 20126 Milan, Italy; m.cordara@campus.unimib.it; 3 Department of Pharmacy, University of Napoli Federico II, 80131 Naples, Italy; mariavittoria.morone@unina.it (M.V.M.); lo.delellis2@gmail.com (L.F.D.L.); angycerqua@libero.it (A.C.); alessandra.baldi.alimenti@gmail.com (A.B.); alessandro.diminno@unina.it (A.D.M.); 4 Anaesthesia and Resuscitation A. U. O. Luigi Vanvitelli, 80138 Naples, Italy; roberto.piccinocchi@policliniconapoli.it; 5 Applied Statistic Unit, Department of Earth and Environmental Sciences, University of Pavia, 27100 Pavia, Italy; roberto.sacchi@unipv.it; 6 Comegen S.C.S., Società Cooperativa Sociale, 80125 Naples, Italy; gpiccino@tin.it; 7 CEINGE-Biotecnologie Avanzate, 80145 Naples, Italy; 8 Department of Advanced Biomedical Sciences, University of Naples Federico II, 80131 Naples, Italy; 9 International Research Center for Food Nutrition and Safety, Jiangsu University, Zhenjiang 212013, China

**Keywords:** nutraceuticals, psychometric assessment, mental well-being, food-derived bioactives, aging brain

## Abstract

Background/Objectives: Mild cognitive impairment (MCI) may precede dementia, and safe nutritional strategies able to support cognitive function are of clinical interest. Dietary nucleotides may contribute to membrane phospholipid synthesis, synaptic function, and neuroprotective pathways; however, clinical evidence in older adults with MCI remains limited. This randomized placebo-controlled trial evaluated the efficacy and tolerability of nucleotide-rich yeast extracts from *Kluyveromyces fragilis* and *Saccharomyces cerevisiae*. Methods: Seventy-two participants (mean age 73.5 ± 7.7 years; range 60–85) were randomly assigned (1:1:1) to receive *K. fragilis* extract, *S. cerevisiae* extract, or placebo once daily for 180 days. Cognitive outcomes were assessed using the Montreal Cognitive Assessment (MoCA) and Mini-Mental State Examination (MMSE) at baseline (T0), 90 days (T1), and 180 days (T2); quality of life was assessed using the SF-12 questionnaire at T0 and T2. Treatment effects were analyzed using linear mixed-effects models adjusted for age and sex. Results: After 180 days, MoCA scores increased by 4.42 points in the *K. fragilis* group and 3.92 points in the *S. cerevisiae* group, compared with 0.58 points in the placebo group (time × treatment *p* < 0.001; T0–T2 within-group *p* < 0.001 for both active groups and *p* = 0.14 for placebo). MMSE scores increased by 1.62 and 3.11 points in the *K. fragilis* and *S. cerevisiae* groups, respectively, compared with 0.25 points in the placebo group (time × treatment *p* < 0.001; T0–T2 within-group *p* < 0.001 for both active groups and *p* = 0.57 for placebo). The SF-12 mental component score increased by 7.50 and 9.16 points in the two active groups, respectively (time × treatment *p* = 0.022; T0–T2 *p* = 0.0013 and *p* < 0.001, respectively), while physical quality-of-life scores did not change significantly (PCS time × treatment *p* = 0.11). No adverse events were reported. Conclusions: Nucleotide-rich *K. fragilis* and *S. cerevisiae* yeast extracts were well tolerated and were associated with improved cognitive scores over six months in older adults with MCI. Larger multicenter trials are needed to confirm these findings.

## 1. Introduction

Dementia is a progressive neurodegenerative disorder characterized by cognitive decline that significantly impairs the ability to perform daily activities. Rather than representing a single disease, dementia is an umbrella term encompassing a group of conditions that lead to deterioration in cognitive function [1]. It represents a major global public health challenge, currently affecting approximately 47 million people worldwide, with the number expected to increase to 131 million by 2050 [2]. In the United States and other developed countries, the age-adjusted incidence of dementia has declined over the past two decades, possibly due to improved levels of formal education. Nevertheless, in the absence of effective preventive or therapeutic strategies, the overall burden and adverse consequences of dementia are expected to continue rising [3].

Dementia can be broadly classified into reversible and irreversible forms. Reversible dementia may result from potentially treatable conditions such as Obstructive sleep apnea, vitamin B12 deficiency, Major depressive disorder, polypharmacy and misuse of sedative or anticholinergic medications; in these cases, cognitive impairment is secondary and may improve or resolve once the underlying pathology is treated. In contrast, most cases of dementia are irreversible and can be categorized into primary (i.e., Alzheimer’s disease, frontotemporal dementia, and dementia with Lewy bodies) [4,5] and secondary (i.e., vascular dementia) forms. Beyond cognitive impairment, dementia is frequently accompanied by a range of neuropsychiatric and behavioral symptoms, including anxiety, depression, and personality changes [6].

In its early stages, dementia is often preceded by mild cognitive impairment (MCI), a clinical condition characterized by a modest decline in one or more cognitive domains while functional independence in daily activities is largely preserved. Importantly, MCI does not inevitably progress to dementia. Individuals diagnosed with MCI may remain stable over time, revert to normal cognitive status, or progress to dementia [7,8,9,10]. Several risk factors have been associated with the onset and progression of MCI, including alcohol consumption, smoking, obesity, diabetes, hypertension, hypercholesterolemia, and poor dietary habits [11].

Given that MCI does not necessarily progress to dementia, early intervention targeting modifiable risk factors is crucial to prevent or delay cognitive decline. Such strategies aim to preserve cognitive function, maintain independence in daily activities, and mitigate behavioral symptoms [12]. In the early stages of cognitive decline, interventions typically include cognitive rehabilitation and physical exercise, both of which have shown beneficial effects in improving cognitive performance, maintaining autonomy, and reducing depressive symptoms. Nutritional supplementation is also commonly considered, particularly for potentially deficient nutrients or endogenous compounds, including vitamins (e.g., vitamin E and folic acid), omega-3 fatty acids, minerals (e.g., selenium), nucleotides, and bioactive compounds such as those occurring in plant extracts (e.g., *Panax ginseng*, *Ginkgo biloba*, and *Eleutherococcus*) [13,14,15,16,17,18].

Among nutritional interventions, nucleotide supplementation has emerged as a promising approach. Nucleotides are low-molecular-weight molecules composed of a pentose sugar, a nitrogenous base, and one or more phosphate groups. They play essential roles in cellular energy metabolism, immune regulation, and tissue repair [19]. Although nucleotides can be synthesized endogenously, certain tissues have a limited capacity for de novo synthesis and therefore rely on exogenous sources, particularly under conditions of increased physiological demand [20,21]. Interest in the potential effects of dietary nucleotides on the nervous system dates to 1995, when Sato and colleagues demonstrated that dietary nucleotide supplementation in Sprague–Dawley rats significantly increased the levels of phosphatidylcholine and phosphatidylethanolamine, two major phosphoglycerides of cell membranes that are essential for maintaining membrane fluidity. Alterations in the balance of these phospholipids have been implicated in several neurodegenerative disorders, including Alzheimer’s disease and Parkinson’s disease [22]. Since then, preclinical and clinical studies have reported promising effects of nucleotide-related compounds such as uridine, citicoline, and nicotinamide riboside in improving cognitive function and mitigating cognitive impairment [23,24,25,26,27,28]. A recent investigation in a non-genetic mouse model of Alzheimer’s disease showed that a commercial *Kluyveromyces fragilis* yeast extract (RiboDIET^®^), rich in nucleotides, lessened oxidative stress, brain inflammation, and amyloid pathology via modulation of iron-related metabolic proteins [29], supporting further investigation of this ingredient as a protective agent against neuroinflammation, a process closely linked to cognitive decline [30]. These data provided a preclinical rationale for testing RiboDIET^®^ in humans, although clinical evidence in older adults with MCI remains limited and comparative data for nucleotide-rich yeast extracts obtained from different yeast species are lacking.

Based on this rationale, the present single-center, randomized, placebo-controlled, double-blind, parallel-arm clinical trial was designed to evaluate the efficacy and tolerability of yeast extracts obtained from *K. fragilis* or *S. cerevisiae*, serving as dietary sources of nucleotides, on cognitive function and quality of life in adults with MCI. In addition, exploratory comparison between both yeast extract groups was performed to evaluate if source of yeast-derived nucleotides differentially influences cognitive outcomes.

## 2. Methods

### 2.1. Clinical Trial Design and Ethics

This randomized, placebo-controlled, parallel-group clinical trial was conducted at COMEGEN Soc. Coop. Sociale (Naples, Italy) to evaluate the efficacy and tolerability of two nucleotide-rich food supplements based on yeast extracts obtained from *K. fragilis* and *S. cerevisiae* in older adults with MCI. The manuscript was prepared in accordance with CONSORT reporting recommendations, and the completed CONSORT 2025 checklist was provided as Supplementary Documentation. A total of 72 participants were enrolled and randomly assigned in a 1:1:1 ratio to receive *K. fragilis* Y.E., *S. cerevisiae* Y.E., or placebo once daily for 180 days. Outcomes were assessed at baseline (T0), after 90 days of treatment (T1), and after 180 days of treatment (T2).

The research was conducted in accordance with the ethical principles of the Declaration of Helsinki (Helsinki 1964 and subsequent amendments up to Fortaleza 2013) and the Guidelines for Good Clinical Practice (CPMP/ICH/135/95). The study protocol was reviewed and approved by the competent Ethics Committee prior to the initiation of the trial (Protocol number 4/25, 8 April 2025). All participants received detailed information regarding the objectives, methodology, and procedures of the clinical trial prior to enrollment and provided written informed consent before any study-related procedure was performed. The consent forms were signed by both the participant and the investigator, and copies were provided to the participants. The informed consent process complied with Regulation (EU) 2016/679 regarding the protection of personal data. This study is listed on the ISRCTN registry under the ID number ISRCTN99002302 (registered on 2 March 2026) [31]. Clinical data were collected and processed anonymously in accordance with applicable privacy regulations.

The study consisted of three scheduled visits. During visit 1 (baseline, T0), participants were screened for eligibility according to the predefined inclusion and exclusion criteria. During this visit, informed consent was obtained, and a fourth-generation HIV rapid saliva test was performed. Subjects with doubtful or positive results were excluded from participation. Eligible subjects were then randomized using a computer-generated randomization sequence prepared by an independent researcher not involved in participant assessment or data analysis. Allocation concealment was ensured using sequentially numbered, identical treatment containers prepared according to the randomization list and dispensed only after completion of baseline assessments. The study was double-blind: participants, investigators, and outcome assessors were unaware of group assignment throughout the intervention period. Baseline assessments included MoCA and MMSE as primary efficacy outcomes and the SF-12 quality-of-life questionnaire as a secondary outcome. During visit 2 (T1, after 90 days), MoCA and MMSE were repeated, compliance was evaluated through collection of product packaging, participants received the next 90-day supply, and adverse events were actively assessed. During visit 3 (T2, after 180 days), MoCA, MMSE, and SF-12 were administered, compliance was verified, and adverse events were again assessed.

During the entire study period, participants were required to attend all scheduled visits at the study center and were instructed not to use medications or dietary supplements that could interfere with the study outcomes unless approved by the investigator. The total duration of the study was approximately 8 months, including 1 month for recruitment, 6 months for treatment, and approximately 1 month for data analysis.

No interim analysis or formal stopping rule was planned because of the limited duration of the study and the expected low-risk profile of the food supplements; nevertheless, safety and tolerability were monitored throughout the trial.

### 2.2. Food Supplements and Placebo

The commercially available yeast extracts RiboDIET^®^ and RiboMIX SC were obtained from Prosol S.r.l. (Madone, Bergamo, Italy) from *K. fragilis* and *S. cerevisiae*, respectively, through a standardized and controlled process that does not use organic solvents. RiboDIET^®^ and RiboMIX SC are food supplement ingredients marketed by Prosol S.r.l.; their dried-extract composition is reported in the Supplementary Material (Tables S1 and S2).

RiboDIET^®^ and RiboMIX SC contain free 5′-monophosphate nucleotides (approximately 40% and 30%, respectively), including adenosine monophosphate (5′AMP), cytidine monophosphate (5′CMP), uridine monophosphate (5′UMP), and guanosine monophosphate (5′GMP), along with nucleosides, oligonucleotides, ribonucleic acid fragments, amino acids, minerals, and B-group vitamins. For the clinical trial, the two supplements were formulated as gastro-resistant capsules to provide a comparable nucleotide dose. Each *K. fragilis* Y.E. capsule contained 250 mg of yeast extract, 150 mg of maltodextrin, 5 mg of magnesium salts of fatty acids, and a 95 mg gastro-resistant capsule shell. Each *S. cerevisiae* Y.E. capsule contained 350 mg of yeast extract, 50 mg of maltodextrin, 5 mg of magnesium salts of fatty acids, and a 95 mg gastro-resistant capsule shell. The placebo consisted of 390 mg of maltodextrin, 15 mg of magnesium salts of fatty acids, and a 95 mg gastro-resistant capsule shell, and was identical in shape, color, odor, taste, and packaging to the active treatments to maintain blinding.

Participants were instructed to take one capsule of the assigned food supplement (*K. fragilis* Y.E. or *S. cerevisiae* Y.E.) or placebo daily, preferably between meals, with a small amount of water. In accordance with European legislation, participants were advised not to exceed the recommended daily dose, that the product was not intended as a substitute for a balanced diet and healthy lifestyle, and to store the product in a cool, dry place away from heat sources. The food supplements and placebo were manufactured by FMC S.r.l. (Ferentino, Frosinone, Italy) in accordance with European specifications for contaminants and microbiological limits.

The two food supplements were notified to the Italian Ministry of Health (*K. fragilis* Y.E. notification number: 197263; *S. cerevisiae* Y.E. notification number: 197264) in accordance with Directive 2002/46/EC on food supplements and the Italian Ministry of Health guidelines [32], which require clinical studies on foods and food ingredients to focus on products that comply with existing food regulations. The study products were supplied free of charge by Prosol S.r.l. (Madone, Bergamo, Italy), sponsor of the clinical trial.

### 2.3. Inclusion and Exclusion Criteria

Participants of both sexes were eligible for inclusion if they met all of the following criteria: age between 60 and 85 years; ability to understand and sign informed consent; ability to comply with study protocol requirements; negative HIV test; MoCA score between 20 and 25, suggesting MCI; total score of 14 in Instrumental Activities of Daily Living (IADL) and Activities of Daily Living (ADL), suggesting full functional independence; Geriatric Depression Scale (GDS) score between 0 and 5, suggesting absence of depressive symptoms; Generalized Anxiety Disorder-7 (GAD-7) score between 0 and 4, suggesting absence of anxious symptoms; no current use of medications affecting the nervous system, including antidepressants, anxiolytics, opiates, or antidiabetic drugs; and no antibiotic treatment within the previous four weeks.

Participants were excluded if, despite otherwise meeting the inclusion criteria, they had difficulty collaborating or attending scheduled visits; medical conditions considered incompatible with participation, including active systemic diseases, diabetes, neurological disorders, or psychiatric disorders; HIV infection or immunodeficiency; severe visual or hearing impairment; known allergy to any ingredient of the investigational products; substance abuse involving alcohol, drugs, nicotine, caffeine, or theine; or current use of medications considered incompatible with the study protocol.

### 2.4. Efficacy Outcomes

The primary efficacy outcome was the change in cognitive function after supplementation with yeast-extract-based food supplements. The efficacy parameters were the MoCA and MMSE scores measured at baseline (T0), after 90 days (T1), and after 180 days (T2). The MoCA is a rapidly administrable neuropsychological test scored out of 30 points, with higher scores suggesting better performance. It examines visuospatial/executive function, naming, episodic memory, attention, language, abstraction, and orientation [33]. A score below 26 suggests mild signs of cognitive impairment, whereas a score below 20 may be indicative of dementia and requires further assessment. The MMSE is a 30-point quantitative psychometric test that evaluates time orientation, place orientation, registration, attention and calculation, recall, naming, repetition, complex command, reading, and praxis. The total score ranges from 0, indicating maximum cognitive deficit, to 30, indicating no cognitive deficit [34]. Raw MMSE scores were corrected for age and education according to tabulated values; a corrected score above 24 is considered normal, whereas subjects with dementia usually have a score below 18 [35].

The secondary efficacy outcome was the change in health-related quality of life, assessed using the SF-12 questionnaire. The SF-12 is a validated 12-item self-reported instrument that measures eight domains: physical functioning, role-physical, bodily pain, general health, vitality, social functioning, role-emotional, and mental health. The questionnaire yields a Physical Component Summary (PCS) score and a Mental Component Summary (MCS) score and was administered at baseline (T0) and after 180 days of treatment (T2) [36].

### 2.5. Safety and Tolerability

The investigated food supplements consisted of yeast extracts derived from *K. fragilis* and *S. cerevisiae*, ingredients commonly used in food supplements and permitted for use in the European Union. On the basis of their regulatory status and available safety information, no specific safety signal was anticipated; nevertheless, safety and tolerability were prespecified because clinical exposure in older adults with MCI required systematic documentation. Participants were monitored at each visit and were instructed to report any discomfort or adverse event at any time. In the event of suspected adverse reactions, the events would be reported through the national VigiErbe phytovigilance system [37] managed by the National Institute of Health (Italy).

### 2.6. Data Collection

Data were collected using structured Case Report Forms (CRFs) specifically designed for the study. The CRFs consisted of two main sections. The first section recorded participant information including personal data, medical history, concomitant medications, and treatment group assignment. The second section recorded study outcomes including results of the cognitive assessments (MoCA and MMSE) and the quality-of-life questionnaire (SF-12). Adverse events were recorded using a dedicated reporting form based on the template provided by the Italian National Institute of Health for suspected adverse reactions related to food supplements.

### 2.7. Statistical Analysis

Power analysis was conducted assuming a balanced design with equal numbers of participants in each group. Based on a medium–small effect size (Cohen’s f = 0.20), a significance level α = 0.05, and power (1 − β) = 0.95, a minimum sample size of 66 participants was estimated. Considering a potential dropout rate of approximately 10%, the final planned sample size was increased to 72 participants, corresponding to 24 subjects per group. Baseline characteristics were compared across groups using one-way ANOVA for continuous variables and chi-square tests for categorical variables.

To assess treatment effects, linear mixed-effects models (LMMs) with random intercepts were applied. In these models, the scores represented the dependent variables, while measurement time points (T0, T1, T2), treatment group (placebo, *K. fragilis* Y.E., and *S. cerevisiae* Y.E.), and their interaction were included as fixed effects. Age and sex were also included as fixed effects to control for potential confounding factors. Subject identity was treated as a random factor to account for inter-individual variability in treatment response.

Descriptive statistics were calculated for each variable, including mean values, standard deviations, and minimum–maximum ranges. The primary analysis was performed according to the intention-to-treat (ITT) principle, including all randomized participants with available outcome data in the groups to which they were assigned. Because no participant withdrew and all primary outcome data were available, the ITT population comprised 72 participants. A per-protocol (PP) sensitivity analysis was also planned, based on participants with adequate adherence, to assess robustness. Missing outcome data would have been handled within the LMM framework under a missing-at-random assumption; no imputation was required. All statistical analyses were performed using the lme4 package [38] in R ver. 4.0.1 [39].

## 3. Results

**Figure 1:**
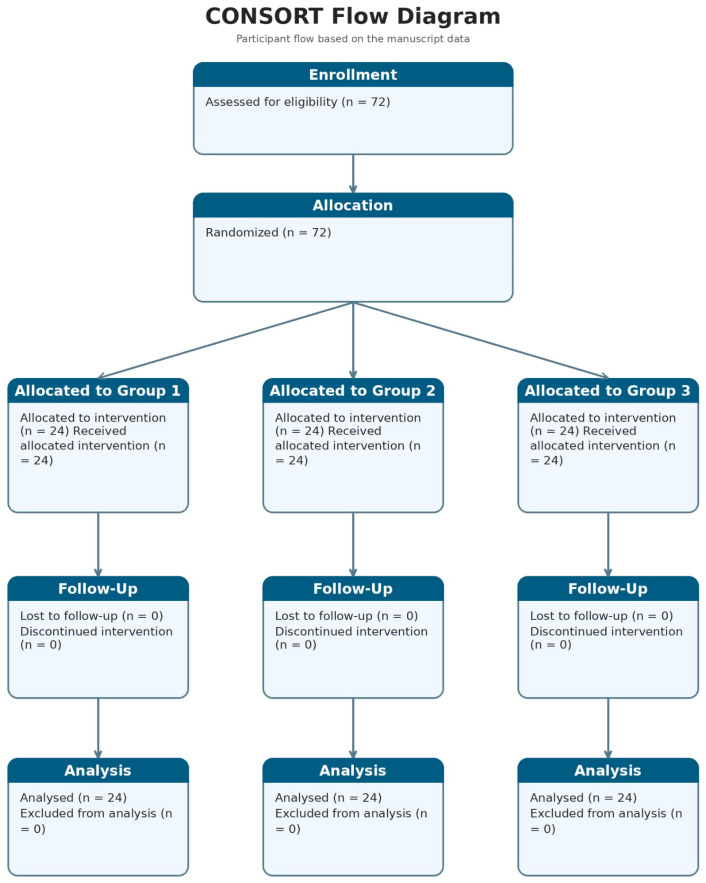
CONSORT flow diagram.

Figure 1 displays the study flowchart prepared according to CONSORT reporting guidelines [40]. Table 1 presents baseline demographic and selected clinical/cognitive characteristics of the enrolled participants. The sample included 72 subjects divided into three experimental groups of 24 participants each. The *K. fragilis* Y.E. group consisted of 15 men and 9 women with a mean age of 75.7 ± 6.5 years. The *S. cerevisiae* Y.E. group included 10 men and 14 women with a mean age of 71.2 ± 8.0 years. The placebo group consisted of 13 men and 11 women with a mean age of 73.5 ± 8.2 years. The overall sample had a mean age of 73.5 ± 7.7 years (range 60–85). No statistically significant baseline imbalance was detected for age (*p* = 0.15), sex (*p* = 0.35), educational attainment (*p* = 0.71), baseline MoCA (*p* = 78), baseline MMSE (*p* = 0.75), SF-12 PCS (*p* = 0.98), or SF-12 MCS (*p* = 0.55).

Each participant reported the level of education in order to correct the cognitive scores of the MoCA and MMSE psychometric tests according to the number of years of schooling. These psychometric tests constituted the primary outcomes of the study and were used to assess cognitive function and its evolution over time after the food supplements or placebo treatment. In addition, the SF-12 questionnaire was administered to evaluate physical and mental quality of life. Table 2 reports the descriptive statistics (mean, standard deviation, and range) for the psychometric test and questionnaire scores measured across the different time points (T0, T1, and T2). Moreover, Table 2 reports the compliance level (expressed as number and % of subjects with high, moderate, and low adherence level) in the three experimental groups, which resulted to be high ranging from 79% in the placebo group to 92% in the *K. fragilis* Y.E group). Each participant reported educational level, which was used to correct the cognitive scores of the MoCA and MMSE psychometric tests according to years of schooling. These psychometric tests constituted the primary outcomes of the study and were used to assess cognitive function and its evolution over time after food supplement or placebo treatment. In addition, the SF-12 questionnaire was administered to evaluate physical and mental quality of life. Table 2 reports descriptive statistics (mean, standard deviation, and range) for the psychometric test and questionnaire scores measured across the different time points (T0, T1, and T2). Table 2 also reports compliance level, expressed as the number and percentage of subjects with high, moderate, and low adherence in the three experimental groups; compliance was generally high, ranging from 79% in the placebo group to 92% in the *K. fragilis* Y.E. group. The ITT analysis included all 72 randomized participants. The PP sensitivity analysis, excluding the single participant with low adherence, did not materially change the direction of the estimates or the interpretation of the main findings.

The statistical analysis reported below refers to the ITT population unless otherwise stated and was performed using LMMs to evaluate the effects of treatment and measurement time on the psychometric test and questionnaire scores.

**Figure 2:**
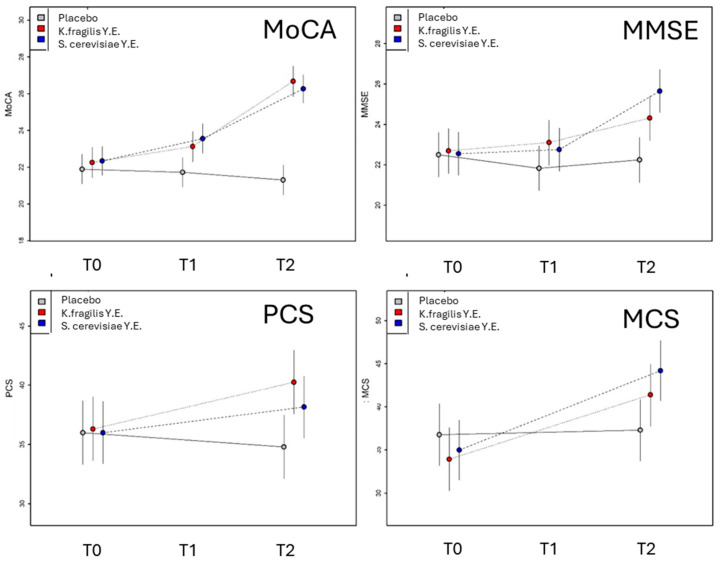
Change between successive measurements of the psychometric tests, MoCA, MMSE, and SF-12 questionnaire scores in the two components PCS and MCS, in the three experimental groups as predicted by LMMs (means and 95% confidence intervals).

The LMM for the MoCA score (Table 3) identified significant effects for measurement (*p* < 0.001), treatment (*p* < 0.001), and their interaction (*p* < 0.001). No significant effects were observed for age or sex (Table 3). These results indicate that MoCA scores varied over time differently across the three experimental groups. At baseline (T0), the MoCA scores did not differ significantly between treatments (β < 0.46 ± 0.52, t_145_ < 0.883, *p* > 0.38). In the *K. fragilis* Y.E. group, the MoCA score increased slightly but significantly between T0 and T1 (β = 0.87 ± 0.39, t_138_ = 2.254, *p* = 0.026) and increased markedly between T1 and T2 (β = 3.54 ± 0.39, t_138_ = 9.126, *p* < 0.001). Consequently, the increase between T0 and T2 was also statistically significant (β = 4.42 ± 0.39, t_138_ = 11.38, *p* < 0.001). A similar trend was observed in the *S. cerevisiae* Y.E. group, with a significant increase between T0 and T1 (β = 1.21 ± 0.39, t_138_ = 3.114, *p* = 0.0022) and between T1 and T2 (β = 2.71 ± 0.39, t_138_ = 3.476, *p* < 0.001). Consequently, the increase between T0 and T2 was also statistically significant (β = 3.92 ± 0.39, t_138_ = 4.684, *p* < 0.001). The MoCA scores of the *K. fragilis* Y.E. and *S. cerevisiae* Y.E. groups did not differ significantly at T1 (β = 0.41 ± 0.53, t_145_ = 0.788, *p* = 0.43) or at T2 (β = 0.42 ± 0.53, t_142_ = 0.794, *p* = 0.43). In the placebo group, MoCA scores did not vary significantly between successive measurements (β < 0.42 ± 0.39, t_138_ < 1.074, *p* > 0.28), and consequently no significant difference was observed between T0 and T2 (β = 0.58 ± 0.39, t_138_ = 1.503, *p* = 0.14). The MoCA score of the placebo group was significantly lower than that of the supplement-treated groups at both T1 (β > 1.14 ± 0.52, t_146_ > 2.738, *p* < 0.0070) and T2 (β > 4.96 ± 0.52, t_145_ > 9.556, *p* < 0.001) (Figure 2).

The LMM for the MMSE questionnaire score (Table 3) identified significant effects for measurement (*p* < 0.001), treatment (*p* = 0.036), and their interaction (*p* < 0.001). A significant effect of age was also observed (*p* = 0.0033), whereas no effect of sex was detected (Table 3). These results indicate that MMSE scores changed differently over time across the three experimental groups (Figure 2). At baseline (T0), MMSE scores did not differ significantly between treatments (β < 0.19 ± 0.69, t118 < 0.270, *p* > 0.79). In the *K. fragilis* Y.E. group, the MMSE score remained stable between T0 and T1 (β = 0.42 ± 0.44, t_138_ = 0.945, *p* = 0.35), but increased significantly between T1 and T2 (β = 1.21 ± 0.44, t_138_ = 2.741, *p* = 0.0069). Consequently, the increase between T0 and T2 was also statistically significant (β = 1.62 ± 0.44, t_138_ = 2.497, *p* < 0.001). In the *S. cerevisiae* Y.E. group, a similar pattern was observed: no significant change between T0 and T1 (β = 0.21 ± 0.44, t_138_ = 0.473, *p* = 0.64), followed by a significant increase between T1 and T2 (β = 2.90 ± 0.44, t_138_ = 6.573, *p* < 0.001). Consequently, the increase between T0 and T2 was also statistically significant (β = 3.11 ± 0.44, t_138_ = 3.977, *p* < 0.001). The MMSE scores of the *K. fragilis* Y.E. and *S. cerevisiae* Y.E. groups did not differ significantly at T1 (β = 0.34 ± 0.70, t_115_ = 0.486, *p* = 0.63), whereas at T2 the value measured in the *S. cerevisiae* Y.E. group tended to be higher than that observed in the *K. fragilis* Y.E. group, although the difference was close to the threshold of statistical significance (β = 1.34 ± 0.70, t_115_ = 1.909, *p* = 0.059). In the placebo group, the MMSE score did not vary significantly between successive measurements (β < 0.67 ± 0.44, t_138_ < 1.512, *p* > 0.13), and no significant difference was observed between T0 and T2 (β = 0.25 ± 0.44, t_138_ = 0.567, *p* = 0.57). The MMSE score of the placebo group was not significantly different from those observed in the supplement-treated groups at T1 (β < 1.27 ± 0.69, t_118_ < 1.837, *p* > 0.069), but it was significantly lower at T2 (β > 2.06 ± 0.69, t_118_ > 2.982, *p* < 0.0035). Regardless of treatment and measurement time, as expected, MMSE scores decreased significantly with increasing age (β = 0.78 ± 0.26, t_67_ = 3.046, *p* = 0.0033).

The LMM for the Physical Component Summary (PCS) score of the SF-12 questionnaire (Table 3) did not identify any significant effects. Therefore, PCS scores did not vary significantly between measurements or among experimental groups (Figure 2).

The LMM for the Mental Component Summary (MCS) score of the SF-12 questionnaire (Table 3) identified significant effects for measurement (*p* < 0.001) and for the interaction between measurement and treatment (*p* = 0.022). No significant effects were observed for treatment alone, age, or sex (Table 3). These results indicate that the MCS score changed over time differently among the three experimental groups (Figure 2). At baseline (T0), MCS scores did not differ significantly between treatments (β < 2.84 ± 2.30, t136 < 1.232, *p* > 0.22). In the groups treated with the supplements, the MCS score increased significantly between T0 and T2 (*K. fragilis* Y.E.: β = 7.50 ± 2.30, t136 = 3.264, *p* = 0.0013; *S. cerevisiae* Y.E.: β = 9.16 ± 2.30, t136 = 3.986, *p* < 0.001), whereas it remained essentially unchanged in the placebo group (β = 0.57 ± 2.30, t136 = 0.247, *p* = 0.80). At T2, the MCS score of the placebo group tended to be lower than that of the *K. fragilis* Y.E. group, although the difference was close to the significance threshold (β = 4.09 ± 2.31, t136 = 1.773, *p* = 0.078). Conversely, the MCS score of the placebo group was significantly lower than that observed in the *S. cerevisiae* Y.E. group (β = 6.86 ± 2.31, t136 = 2.966, *p* = 0.0036) (Figure 2).

## 4. Discussion

In this single-center, randomized, placebo-controlled, parallel-arm, double-blind clinical trial, two food supplements based on yeast extract from *K. fragilis* and *S. cerevisiae* were used as dietary sources of nucleotides to evaluate their efficacy and tolerability on older adults with MCI. Cognitive assessment was performed using the MoCA, a rapidly administered neuropsychological test that assesses visuospatial/executive function, naming, episodic memory, attention, language, abstraction, and orientation [33]. According to the literature, MoCA can detect MCI with high sensitivity [41]. In addition to MoCA, we also used the MMSE, a 30-point quantitative psychometric test for the assessment of cognitive function that evaluates time orientation, place orientation, registration, attention (calculation), recall, naming, repetition, complex command, and reading. This psychometric test, commonly used to evaluate cognitive functions in individuals with severe cognitive decline, such as Alzheimer’s disease patients, was used alongside the MoCA to confirm whether the supplementation resulted in measurable improvements in cognitive functions [34,35].

The results showed that the supplementation with the food supplements for six months significantly improved cognitive function, as reflected by improvement in cognitive test scores. At the baseline, the enrolled subjects showed MCI with MoCA scores ≈ 22, with no significant difference between food supplement and placebo groups, suggesting a correct randomization. After six months of treatment, in the treated groups (*K. fragilis* Y.E. and *S. cerevisiae* Y.E.) the supplementation improved MoCA scores by 4.4 and 3.9 for *K. fragilis* Y.E. and *S. cerevisiae* groups, respectively, exceeding the threshold level of 26 that characterizes subjects with normal cognition. Modest but significant improvement was observed for MMSE score (i.e., 1.6 for *K. fragilis* Y.E. group and 3.1 for *S. cerevisiae* Y.E. group). No significant difference was observed among both treated groups for MoCA score, while regarding MMSE, *S. cerevisiae* Y.E. group showed a trend towards greater improvement.

The observed improvement in MoCA and MMSE scores reflect not only transient symptomatic relief but a significant and progressive improvement of cognitive scores after six months of treatment suggesting possible neurobiological effects of the nucleotide-rich food supplements, rather than immediate placebo-like effect. Dietary nucleotides improved cognitive capacity by two main mechanisms, i.e., by supporting phospholipids and synaptogenesis and by boosting cellular biogenesis or redox pathways that may enhance mitochondrial function and offer neuroprotection [16]. Both mechanisms are important to prevent cognitive decline with aging.

Many in vivo preclinical studies suggest the protective effect of nucleotides. Supplementing rodents with uridine, choline, and DHA increased the amount of brain membranes and synaptic proteins, hence these ingredients serve as precursors for membrane phospholipids and newly formed membranes are utilized for synaptogenesis, underlining enhanced synaptic function as evidenced by neurotransmission and cognition [42,43]. Chen et al. [44] observed reduced memory and learning impairments in SAMP8 mice fed a diet supplemented with nucleosides and nucleotides. Interestingly, these effects were observed only in aged animals and not in young ones, suggesting that the intervention may be beneficial primarily under conditions of compromised nervous system homeostasis, as occurs during aging. In 2013, Cansev et al. [23] demonstrated the neuroprotective effects of uridine in vivo using a neonatal hypoxic–ischemic encephalopathy model and proposed its potential use in infants, given the molecule’s safety and bioavailability. Goren and colleagues [24] further investigated the long-term cognitive effects of uridine treatment in rats exposed to an experimental insult, concluding that uridine exerts positive effects on memory and learning, likely through the reduction in neural cell apoptosis. Some studies also suggest modulation of gut microbiota and microbial metabolites by dietary nucleotides as possible mechanism in neuronal development, neuroprotection, membrane phospholipid production, improving synaptic plasticity, and neurotransmitter regulation, the role as explained by gut–brain axis [45,46]. Mainly, nucleotides increase the abundance of microbes beneficial to neuronal health such as Romboutsia and Akkermansia as well as short chain fatty acids (SCFAs), primarily butyrate.

Some clinical studies aimed at evaluating the effect of dietary nucleotide supplementation have been published in the last two decades with contradictory results. Unlike our study which distinctly focuses on subjects with MCI, most of the previous research involves patients with moderate to severe cognitive decline. A randomized clinical study demonstrated slowing brain atrophy and improvement of some cognitive measures in prodromal Alzheimer’s disease when treated with uridine/choline/DHA-enriched supplement which indicates that enhanced membrane/synapse formation can translate into visible clinical benefits [47]. Supplementing patients with mild to moderate Alzheimer’s disease with uridine (625 mg/day), DHA (1200 mg/day), and choline (400 mg/day) demonstrated an improvement in cognitive decline [25]. Citicoline (500 mg/day) has been studied in a population of healthy elderly with age-associated memory deficits (50–85 years). After the 12-week intervention, supplemental participants showed significant improvements in episodic and composite memory, compared to those in the placebo group, leading the authors to conclude that citicoline improved overall memory performance [26]. In another study, a combination therapy of oral citicoline, memantine and acetylcholinesetesterase inhibitors showed slight improvement in MMSE score as compared to the control group (memantine and acetylcholinesetesterase inhibitors without citicoline) in older patients suffering from Alzheimer’s disease [27]. To the best of our knowledge, the only study that considered nucleotide supplementation for subjects with MCI was published in 2024, by Orr et al. that showed the effect of nicotinamide riboside supplementation (dose escalation to achieve, and maintain, a final dose of 1 g/day over a 10-week study). The significant increase in the blood concentration of NAD+ (a central cofactor for energy metabolism that improves brain function) and a slight trend of improvement in cognitive function was assessed [28].

Regarding quality of life, used as secondary outcome, both food supplements improved MCS score by 7.5 for *K. fragilis* Y.E. and 9.2 for *S. cerevisiae* Y.E., while no significant changes were observed in PCS score across groups or time. A meta-analysis of 17 studies found that improved cognitive function is associated with better quality of life among adults with MCI. However, the authors concluded that due to the high heterogeneity of the included studies more well-designed cognitive intervention trials are necessary [48]. More recently, Song et al. in 79 adults aged 75 years or older with Korean-MoCA (K-MoCA) less than 22, showed that physical and cognitive functions are important factors that can predict quality of life of older adults with cognitive impairment [49].

Furthermore, our study showed that the supplementation was well tolerated throughout the treatment period, with no adverse events reported. The safety profile of nucleotides is high in healthy subjects as demonstrated by numerous studies [16,50,51].

This work has several strengths and limitations. Its main strength is the randomized, placebo-controlled design, which allowed the efficacy and tolerability of the two food supplements to be assessed using validated tools such as MoCA and MMSE. Cognitive impairment is a mental and neurological condition for which pharmacological treatments may be limited by adverse effects, poor adherence, or modest effectiveness. Nutritional approaches are attractive, but robust clinical evidence remains limited. The present findings therefore support further investigation of nucleotide-based supplementation as a possible strategy for MCI management. Another strength is the 24-week treatment duration, which is longer than many previous dietary nucleotide studies and allowed tolerability to be assessed over a clinically meaningful period. The main limitations are the single-center design, the relatively small sample size, the relatively short intervention period (6 months only), the absence of a post-treatment follow-up period, and the enrollment of older adults with MCI only, which limits generalizability to other populations and prevents conclusions on the effects of longer-term use or on longer-term persistence of the effect. Another potential limitation is the practice effect, whereby individuals with MCI may show improved psychometric scores after repeated exposure to the same tasks. This potential bias was partly addressed by including a placebo group; since all groups underwent the same testing schedule, the divergence observed in the supplement-treated groups is less likely to be explained solely by learning effects.

## 5. Conclusions

In conclusion, this exploratory randomized trial suggests that six-month supplementation with nucleotide-rich *K. fragilis* or *S. cerevisiae* yeast extracts is well tolerated and is associated with improved cognitive scores and mental quality of life in older adults with MCI. These findings are encouraging, but they should not be interpreted as definitive evidence of clinical efficacy. Confirmation in adequately powered, multicenter randomized trials with longer follow-up and more diverse populations is required before firm clinical recommendations can be made.

## Figures and Tables

**Table 1:** Baseline demographic and selected clinical/cognitive characteristics of the study population. Group comparisons were performed using one-way ANOVA (F) for continuous variables and chi-square tests (X^2^) for categorical variables (frequencies).

Characteristic	*K. fragilis* Y.E. Group (*n* = 24)	*S. cerevisiae* Y.E Group (*n* = 24)	Placebo Group (*n* = 24)	F/X^2^	*p* Value
Age (years),	75.7 ± 6.5	71.2 ± 8.0	73.5 ± 8.2	F_2.69_ = 1.92	0.15
mean ± SD	(range 64–85)	(range 60–85)	(range 62–85)		
Sex, *n*				X^2^ = 2.09, d.f. = 2	0.35
Males	15	10	13		
Females	9	14	11		
Ethnicity: Caucasian, *n* (%)	24	24	24	N/A	
educational attainment				X^2^ = 0.67, d.f. = 2	0.71
>12 years	17	19	18		
<12 years	7	5	6		
Baseline MoCA score,	22.3 ± 1.5	22.3 ± 1.6	21.9 ± 1.7	F_2.69_ = 0.25	0.78
mean ± SD					
Baseline MMSE score,	23.0 ± 2.8	22.4 ± 2.9	22.6 ± 2.5	F_2.69_ = 0.28	0.75
mean ± SD					
Baseline SF-12 PCS score,	36.1 ± 5.9	35.9 ± 6.7	35.8 ± 6.1	F_2.69_ = 0.015	0.98
mean ± SD					
Baseline SF-12 MCS score,	33.5 ± 8.4	34.7 ± 7.8	36.4 ± 7.5	F_2.69_ = 0.60	0.55
mean ± SD					

**Table 2:** Descriptive statistics (mean, standard deviation, and range) for MoCA and MMSE scores measured at baseline (T0), after 3 months (T1), and after 6 months (T2) of treatment; SF-12 PCS and MCS scores measured at T0 and T2; and total compliance level (number and percentage of subjects with high, moderate, and low adherence) in the three experimental groups (*K. fragilis* Y.E., *S. cerevisiae* Y.E., and placebo).

GROUP		T0	T1	T2
*K. fragilis* Y.E.	MoCA	22.3 ± 1.5	23.2 ± 1.7	26.7 ± 1.8
		(20–25)	(20–26)	(23–30)
	MMSE	23.0 ± 2.8	23.4 ± 2.5	24.6 ± 1.9
		(17.4–28.4)	(19–27.4)	(22–28.4)
	PCS	36.1 ± 5.9		40 ± 5.8
		(25.5–47.2)		(31.2–50.8)
	MCS	33.5 ± 8.4		41 ± 6.6
		(17.3–51.1)		(27.4–50.2)
Compliance		High Adherence	Moderate Adherence	Low Adherence
		0–4 missed capsules	6–10 missed capsules	>10 missed capsules
N° subjects		22	2	0
%		92%	8%	-
*S. cerevisiae* Y.E.	MoCA	22.3 ± 1.6	23.5 ± 2	26.2 ± 1.7
		(20–25)	(20–28)	(24–29)
	MMSE	22.4 ± 2.9	22.6 ± 2.5	25.5 ± 2
		(18–29.3)	(19–29.3)	(22–30.1)
	PCS	35.9 ± 6.7		38 ± 5.7
		(19.5–44.7)		(26.6–48.2)
	MCS	34.7 ± 7.8		43.8 ± 6.6
		(13.7–50)		(29.5–54.8)
Compliance		High Adherence	Moderate Adherence	Low Adherence
		0–4 missed capsules	6–10 missed capsules	>10 missed capsules
N° subjects		21	3	0
%		88%	12%	-
Placebo	MoCA	21.9 ± 1.7	21.7 ± 2.1	21.3 ± 2
		(20–25)	(17–25)	(18–25)
	MMSE	22.6 ± 2.5	21.9 ± 2.1	22.3 ± 3
		(18.2–28)	(17.2–25.3)	(16.2–26.9)
	PCS	35.8 ± 6.1		34.6 ± 5.9
		(22.9–46.8)		(23.5–43)
	MCS	36.4 ± 7.5		37 ± 10
		(21.5–50.7)		(19.2–57.2)
Compliance		High Adherence	Moderate Adherence	Low Adherence
		0–4 missed capsules	6–10 missed capsules	>10 missed capsules
N° subjects		19	4	1
%		79%	16%	4%

**Table 3:** Results of LMMs for treatment comparison (placebo, *K. fragilis* Y.E. and *S. cerevisiae* Y.E. groups) of the scores of the psychometric tests and the questionnaire used in the study at the different measurements (T0, T1 and T2). Abbreviations: df, degrees of freedom; LMM, linear mixed model.

Variable	F	df	*p*-Value
**MoCA**			
Time	72.13	2138	**<0.001**
Treatment	23.27	267	**<0.001**
Age	0.702	167	0.40
Gender	0.005	167	0.94
Time × Treatment	27.56	4138	**<0.001**
**MMSE**			
Time	23.17	2138	**<0.001**
Treatment	3.483	267	**0.036**
Age	9.276	167	**0.0033**
Gender	0.039	167	0.84
Time × Treatment	8.172	4138	**<0.001**
**PCS**			
Time	2.658	169	0.11
Treatment	2.643	267	0.079
Age	0.000	167	0.99
Gender	0.119	167	0.73
Time × Treatment	2.307	269	0.11
**MCS**			
Time	18.73	1136	**<0.001**
Treatment	1.295	2136	0.277
Age	0.063	1136	0.803
Gender	0.221	1136	0.639
Time × Treatment	3.934	2136	**0.022**

## Data Availability

The original contributions presented in this study are included in the article. Further enquiries can be directed to the corresponding author.

## Supplementary Materials

The following supporting information can be downloaded at: https://www.mdpi.com/article/10.3390/nu18121869/s1, Table S1. Concentrations of amino acids, vitamins, and metals of Kluyveromyces fragilis extract (RiboDIET^®^). Table S2. Concentrations of amino acids, vitamins, and metals of Saccharomyces cerevisiae extract (RiboMIX SC).

## Abbreviations

The following abbreviations are used in this manuscript:

ADL	Activities of Daily Living
GAD-7	Generalized Anxiety Disorder-7
GDS	Geriatric Depression Scale
IADL	Instrumental Activities of Daily Living
LMM	Linear Mixed Model
MCI	Mild cognitive impairment
MCS	Mental Component Summary
MMSE	Mini-Mental State Examination
MoCA	Montreal Cognitive Assessment
PCS	Physical Component Summary
SCFAs	Short chain fatty acids
