# Supplementary material for: Effects of Nucleotide-Rich Kluyveromyces fragilis and Saccharomyces cerevisiae Yeast Extracts on Cognitive Function in Older Adults with Mild Cognitive Impairment: A Randomized Placebo-Controlled Trial
Source: Nutrients. 2026 Jun 10;18(12):1869. doi: 10.3390/nu18121869 (PMC13304835; doi:10.3390/nu18121869)
Supplement: Supplementary file 1 [file nutrients-18-01869-s001.zip › nutrients-4323625-supplementary.pdf]

Supplementary Materials

Table S1. Concentrations of amino acids, vitamins, and metals of Kluyveromyces fragilis extract (RiboDIET®).

Nutrient	Concentration	Limit of quantification
**Amino acids**		
Alanine (%)	0.709 ± 0.092	0.05
Arginine (%)	0.42 ± 0.06	0.05
Aspartic acid (%)	1.24 ± 0.16	0.05
Cyst(e)ine, determined as cysteic acid (%)	0.061 ± 0.012	0.05
Glutamic acid (%)	1.94 ± 0.25	0.05
Glycine (%)	1.96 ± 0.25	0.05
Histidine (%)	0.229 ± 0.046	0.05
Isoleucine (%)	0.40 ± 0.06	0.05
Leucine (%)	0.696 ± 0.091	0.05
Lysine (%)	0.93 ± 0.12	0.05
Methionine, determined as methionine sulfone (%)	0.082 ± 0.016	0.05
Phenylalanine (%)	0.38 ± 0.06	0.05
Proline (%)	0.33 ± 0.06	0.05
Serine (%)	0.512 ± 0.067	0.05
Threonine (%)	0.511 ± 0.066	0.05
Tryptophan (%)	0.120 ± 0.024	0.02
Tyrosine (%)	0.184 ± 0.037	0.05
Valine (%)	0.481 ± 0.062	0.05
Sum amino acids (%)	11.19	0.02
**Vitamins**		
Vitamin B1 (calc. as thiamine chloride-HCl) (mg/100 g)	0.061 ± 0.018	0.02
Vitamin B2 (HPLC, calc. as riboflavin) (mg/100 g)	4.07 ± 0.61	0.05
Vitamin B6 (calc. as pyridoxine-HCl) (mg/100 g)	0.48 ± 0.15	0.05
Vitamin B12 (based on cyanocobalamine) (µg/100 g)	58 ± 13	0.3
Niacin (calculated as nicotinic acid) (mg/100 g)	6.4 ± 1.2	0.08
Pantothenic acid (based on D-pantothenic acid) (mg/100 g)	0.379 ± 0.083	0.05
Folic acid (µg/100 g)	1130	5
**Metals**		
Copper (Cu) (mg/kg)	<2.50	5
Iron (Fe) (mg/kg)	121	5
Magnesium (Mg) (%)	0.23	0.01
Manganese (Mn) (mg/kg)	157	5
Mercury (Hg) (mg/kg)	<0.05	0.05
Potassium (K) (%)	0.26	0.01
Selenium (Se) (mg/kg)	<0.10	0.1
Sodium (Na) (%)	6.2	0.01
Arsenic (As) (mg/kg)	<0.20	0.2
Lead (Pb) (mg/kg)	<0.20	0.2
Chromium (Cr) (mg/kg)	0.42	0.15
Tin (Sn) (mg/kg)	<1.00	1
Chloride (mg/100 g)	1610	100

Table S2. Concentrations of amino acids, vitamins, and metals of Saccharomyces cerevisiae extract (RiboMIX SC).

Nutrient	Concentration	Limit of quantification
**Amino acids**		
Alanine (%)	2.18 ± 0.28	0.05
Arginine (%)	1.68 ± 0.22	0.05
Aspartic acid (%)	4.04 ± 0.40	0.05
Cyst(e)ine, determined as cysteic acid (%)	0.258 ± 0.052	0.05
Glutamic acid (%)	6.50 ± 0.65	0.05
Glycine (%)	2.87 ± 0.37	0.05
Histidine (%)	0.80 ± 0.10	0.05
Isoleucine (%)	1.47 ± 0.19	0.05
Leucine (%)	2.16 ± 0.28	0.05
Lysine (%)	2.85 ± 0.37	0.05
Methionine, determined as methionine sulfone (%)	0.34 ± 0.06	0.05
Phenylalanine (%)	1.37 ± 0.18	0.05
Proline (%)	1.36 ± 0.18	0.05
Serine (%)	1.42 ± 0.18	0.05
Threonine (%)	1.47 ± 0.19	0.05
Tryptophan (%)	0.528 ± 0.069	0.02
Tyrosine (%)	1.11 ± 0.14	0.05
Valine (%)	1.74 ± 0.23	0.05
Sum amino acids (%)	34.15	0.9324
**Vitamins**		
Vitamin B1 (calc. as thiamine chloride-HCl) (mg/100 g)	0.077 ± 0.023	0.02
Vitamin B2 (HPLC, calc. as riboflavin) (mg/100 g)	4.12 ± 0.62	0.05
Vitamin B6 (calc. as pyridoxine-HCl) (mg/100 g)	1.59 ± 0.48	0.05
Vitamin B12 (based on cyanocobalamine) (µg/100 g)	18.1 ± 4.2	0.3
Niacin (calculated as nicotinic acid) (mg/100 g)	22.3 ± 4.0	0.08
Pantothenic acid (based on D-pantothenic acid) (mg/100 g)	1.00 ± 0.22	0.05
**Metals**		
Copper (Cu) (mg/kg)	9.43	5
Iron (Fe) (mg/kg)	24.3	5
Magnesium (Mg) (%)	0.16	0.01
Manganese (Mn) (mg/kg)	7.73	5
Mercury (Hg) (mg/kg)	<0.05	0.05
Potassium (K) (%)	1.88	0.01
Selenium (Se) (mg/kg)	<0.10	0.1
Sodium (Na) (%)	7.2	0.01
Arsenic (As) (mg/kg)	<0.20	0.2
Lead (Pb) (mg/kg)	<0.20	0.2
Chromium (Cr) (mg/kg)	0.80	0.15
Tin (Sn) (mg/kg)	<1.00	1
Chloride (mg/100 g)	6270	100
**Folates**		
Folic acid (µg/100 g)	459	1
10-Formyl folic acid (µg/100 g)	720	1
10-Methyl folic acid (µg/100 g)	<2.00	2
5-Formyl-5,6,7,8-tetrahydrofolate (µg/100 g)	203	1
5-Methyl-5,6,7,8-tetrahydrofolate (µg/100 g)	<1.00	1
5,6,7,8-Tetrahydrofolate (µg/100 g)	<4.00	4
Total folate, calculated as folic acid (µg/100 g)	1330	0.9324
